# Automatic lesion detection and segmentation of ^18^F-FET PET in gliomas: A full 3D U-Net convolutional neural network study

**DOI:** 10.1371/journal.pone.0195798

**Published:** 2018-04-13

**Authors:** Paul Blanc-Durand, Axel Van Der Gucht, Niklaus Schaefer, Emmanuel Itti, John O. Prior

**Affiliations:** 1 Department of Nuclear Medicine, Henri Mondor University Hospital, Créteil, France; 2 Department of Nuclear Medicine and Molecular Imaging, Lausanne University Hospital, Lausanne, Switzerland; George Washington University, UNITED STATES

## Abstract

**Introduction:**

Amino-acids positron emission tomography (PET) is increasingly used in the diagnostic workup of patients with gliomas, including differential diagnosis, evaluation of tumor extension, treatment planning and follow-up. Recently, progresses of computer vision and machine learning have been translated for medical imaging. Aim was to demonstrate the feasibility of an automated ^18^F-fluoro-ethyl-tyrosine (^18^F-FET) PET lesion detection and segmentation relying on a full 3D U-Net Convolutional Neural Network (CNN).

**Methods:**

All dynamic ^18^F-FET PET brain image volumes were temporally realigned to the first dynamic acquisition, coregistered and spatially normalized onto the Montreal Neurological Institute template. Ground truth segmentations were obtained using manual delineation and thresholding (1.3 x background). The volumetric CNN was implemented based on a modified Keras implementation of a U-Net library with 3 layers for the encoding and decoding paths. Dice similarity coefficient (DSC) was used as an accuracy measure of segmentation.

**Results:**

Thirty-seven patients were included (26 [70%] in the training set and 11 [30%] in the validation set). All 11 lesions were accurately detected with no false positive, resulting in a sensitivity and a specificity for the detection at the tumor level of 100%. After 150 epochs, DSC reached 0.7924 in the training set and 0.7911 in the validation set. After morphological dilatation and fixed thresholding of the predicted U-Net mask a substantial improvement of the DSC to 0.8231 (+ 4.1%) was noted. At the voxel level, this segmentation led to a 0.88 sensitivity [95% CI, 87.1 to, 88.2%] a 0.99 specificity [99.9 to 99.9%], a 0.78 positive predictive value: [76.9 to 78.3%], and a 0.99 negative predictive value [99.9 to 99.9%].

**Conclusions:**

With relatively high performance, it was proposed the first full 3D automated procedure for segmentation of ^18^F-FET PET brain images of patients with different gliomas using a U-Net CNN architecture.

## Introduction

Positron emission tomography (PET) is one of the most advanced medical imaging technologies at the molecular level for human functional imaging. Gliomas constitute the most frequent brain tumors [1]. As ^18^F-Fluorodeoxyglucose shows high background uptake in normal brain, radiolabeled amino acids such as ^11^C-methionine, ^18^F-fluoro-L-dopamine and ^18^F-fluoro-ethyl-tyrosine (^18^F-FET) [2,3] are more appropriate as they exhibit lower background uptake and can therefore depict brain tumors with a higher contrast. These tracers are increasingly used in the diagnostic workup of patients with gliomas, including differential diagnosis, evaluation of tumor extension, treatment planning and follow-up [4]. Even if PET remains the modality of choice for its high sensitivity, it has several limitations including low spatial resolution with partial volume effect and relatively low signal-to-noise ratio. Therefore, brain tumor segmentation is challenging, not only due to the technology limits but also because of the large variations of shape and intensity of brain tumor uptake relating to the large heterogeneity in histology, genetics, and outcome [5].

Segmentation is a common task in medical imaging and can be performed manually, semi-automatically or automatically. It has multiple aims such as the definition of a gross tumor volume or biological tumor volume (BTV) for radiotherapy planning and is necessary for accurate evaluation and monitoring of tumor response. It also has shown to have some independent prognostic value such as metabolic tumor volume for example in patients with diffuse large B-cell lymphoma [6]. One important step of an ^18^F-FET PET interpretation requires the definition of a volume of interest (VOIs), which has a significant impact on diagnosis, staging, radiotherapy planning and treatment assessment. Indeed, analysis of time activity curves (TAC) requires a VOI and may have some interest for tumor grading [7,8], characterizing molecular phenotypes [9] or differentiating radio-necrosis from progressive disease [10]. ^18^F-FET PET VOIs are usually performed by manually drawing a 3D spherical ellipse around the highest uptake, and/or by thresholding at standardized uptake values (SUVs).

Nevertheless, manual delineation is time consuming and subject to intra- and inter-observer variations among readers. Intensity-based thresholding is commonly used for VOI segmentation in PET. Two approaches are used for thresholding, either with a fixed threshold (SUV = 2.5) or an adaptive threshold (e.g. 41% of SUV_max_). Nevertheless, these approaches do not consider the spatial correlations of voxels in an image, making such methods sensitive to image noise, uptake inhomogeneity and partial volume effect. Furthermore, these types of segmentation are also sensitive to the selected threshold values that can lead in large differences of volume [11].

To avoid such problems, some automatic PET segmentation methods have been suggested and recently reviewed by Hatt et al. [12]. Some of these approaches are 2D methods that consider each PET slice individually or 3D methods that seem more appropriate as they consider the contextual information of the whole volume. Main 3D techniques include gradient-based segmentation, region growing (such as fuzzy-c-means), statistical algorithms, machine learning and texture-based segmentation. Recently, progresses in computer vision and machine learning have been translated in medical imaging. Convolutional neural networks (CNN) can gather local information around a voxel and produce a likelihood map, rather than an output for a single voxel [13]. The U-Net architecture comprises a fully connected CNN followed by an upsampling part. Then, aim was to demonstrate the feasibility of an automated ^18^F-FET PET lesion detection and segmentation relying on a full 3D U-Net CNN.

## Materials and methods

### Patients

Thirty-seven patients were retrospectively included. Characteristics are given in S1 Table. Every patient underwent a ^18^F-FET PET/CT at diagnosis before any planned surgical stereotaxic biopsy or treatment (tumor resection, chemotherapy, radiotherapy). Patients who required rapid surgery (<2 weeks) due to mass effect or intracerebral hemorrhage, as well as patients with history of brain biopsy, surgery or brain treatment were excluded. All patients underwent imaging procedures as standard care and gave written informed consent before inclusion. The retrospective analysis was approved by the local Human Research Ethics Committee of the State of Vaud (#2017–00758).

### ^18^F-FET PET acquisition

Patients underwent a dynamic ^18^F-FET PET/CT on Discovery D690 time-of-flight (27 patients) and Discovery LS (10 patients) within the same imaging department (GE Healthcare, Waukesha, WI, USA). They were required to fast for at least 4 hours before ^18^F-FET injection as recommended by international guidelines [14]. After intravenous injection of 214 ± 25 MBq (range 145 to 295 MBq) of ^18^F-FET, PET images were acquired using a dynamic protocol over 50 minutes (10 frames of 5 min; 3.3-mm or 4.2-mm section thickness; 24 cm field-of-view, matrix size of 256 × 256). Calibration for the two machines was the same. ^18^F-FET image volumes were reconstructed by the iterative method ordered-subset expectation maximization (OSEM, 3 iterations and 16 subsets) including a Gaussian post-filter (FWHM = 5 mm).

### ^18^F-FET PET image characteristics and normal distribution

Normal brain distribution of ^18^F-FET demonstrated relatively low background uptake, mainly due to venous drainage in lateral/sagittal sinus or cavernous system and fat (subcutaneous, peri-orbitary). Bordure artifacts were sometimes seen in the limits of the field of view. No false positive was expected from this dataset. Patients were included in a clinical trial and any abnormal uptake within brain white or grey matter was considered as pathologic. CT volumes were not available in any patient and therefore manual segmentation only relied on PET data. Taking into account that the maximum uptake of ^18^F-FET was not necessary the tumor, in order to achieve brain tumor segmentation, it was decided to follow a two-step procedure. First, a detection of the glial tumor using a U-Net CNN, followed by a second part of proper segmentation using morphological dilatation and thresholding.

### Ground truth ^18^F-FET PET segmentation

All dynamic ^18^F-FET PET brain image volumes were temporally realigned to the first dynamic acquisition, coregistered and spatially normalized onto the Montreal Neurological Institute template (McGill University, Montreal, Canada). Dimensions of the resulting voxels were 2x2x2 mm3. A summation image from the 20^th^ to 40^th^ minute post-injection served as reference either for manual segmentation or training. Spatial normalization was performed using the Statistical Parametric Mapping software (SPM 12) implemented in Matlab version R2015a (Mathworks Inc., Sherborn, MA). To perform a semi-automatic contouring, a manually drawn mask around the tumor was performed with LifeX Software (https://www.lifexsoft.com, CEA, Saclay, France) [15,16] and a threshold was set to 1.3 x background as suggested by Bette et al. [17–19], where the background was defined as the mean value of a 10 cm^3^ spherical VOI in the contralateral hemisphere.

### Pre-processing

For computational purposes, all images (masks and summation images) were resized with a linear interpolation to a 64x64x40 volume. Due to the limited resolution of PET and the blurring effects of the point spread function, it was performed a gradient magnitude calculation that was added to the original image to reinforce contour definition. Each volume was normalized from mean and standard deviation. To avoid overfitting, a data-augmentation strategy was used to enlarge the training dataset. This procedure included rotations (–30 and +30°), translations (–0.1, 0.1) shearing (–10, +10°), and zooming (0.85, 1.15). To avoid overfitting and to evaluate the segmentation procedure in clinical conditions, dataset was randomly split between a training group and a validation group (70 and 30% of the whole population, respectively).

### Implementation

The volumetric CNN was implemented based on a modified Keras implementation of a U-Net library supporting 3D operations. All the trainings and experiments were conducted on a personal computer work-station equipped with a NVIDIA TITAN X 1080 Ti graphics processing unit (GPU). The networks were trained with Stochastic Gradient Descent Adam Optimizer method, with Dice Similarity Coefficient (DSC) as an accuracy measure of the segmentation procedure, and minus DSC as a loss function that was backpropagated through the CNN. The batch size was set to 5. The learning rate was set to 10^−4^, initially; the model was trained for up to 150 epochs.

### Architecture of the U-Net

**Fig 1:**
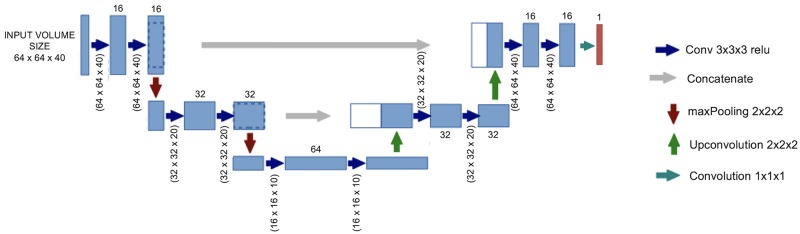
Architecture of the fully connected CNN with a 3 layer “U-Net” architecture.

The network architecture followed as described by Ronneberger et al. [20] is illustrated in Fig 1. It consisted of an encoding path seen at the left side and a decoding path at the right side of Fig 1. The encoding path followed a typical architecture of a CNN with repeated application of two 3×3×3 convolutions (with a padding of 2). Each was followed by a rectified linear unit (ReLU) and a 2×2×2 maxPooling operation with a stride of 2 for downsampling. At each downsampling step, the number of feature channels was doubled, starting with 16 channels at the fist convolution. Every step in the expansion path consisted of: 1) an upsampling of the feature map followed by a 3×3×3 convolution (“up-convolution”) that halved the number of feature channels; 2) a concatenation with the correspondingly cropped feature map from the contracting path; and 3) two 3×3×3 convolutions, each followed by a ReLU. At the final layer, a 1×1×1 convolution was used to map each component feature vector to the desired number of classes (here 2). In total, the network had 11 convolution layers and 387,889 parameters had to be trained. The predicted mask from the U-Net was followed by a morphological dilatation with a 3×3×3 square connectivity. Also, it was performed a fixed thresholding, where the threshold was set to 1.3 times the mean value of an hemispheric swap of the predicted U-Net mask to match the procedure that was performed for ground truth. All these computations were performed using python 2.7 with numpy, nilearn and scikit-learn packages [21].

### Metrics

To assess the performance of the segmentation procedure, a DSC was computed. It was used as an accuracy measure for the training of the U-Net and also as a loss function. DSC was computed as followed where P and *T* corresponded to the prediction of the U-Net model and the ground truth respectively.

$$DSC\;(P,T)=\frac{2*P\cap T}{P+T}$$

Values of sensitivity (Se), specificity (Sp), positive predictive value (PPV) and negative predictive value (NPV) were calculated at the voxel level on the validation set of 11 patients. Se, Sp, PPV and NPV were defined as follow:

$$Se=\frac{TP}{TP+FN}$$

$$Sp=\frac{TN}{TN+FP}$$

$$PPV=\frac{TP}{TP+FP}$$

$$NPV=\frac{TN}{TN+FN}$$

where TP (True Positive) was the number of voxels correctly classified as tumor, TN (True Negative) the number of voxel correctly classified as non-tumor, FP (False Positive) the number of voxels wrongly classified as tumor and FN (False Negative) the number of voxels patients wrongly classified as non-tumor.

## Results

### Detection

Thirty-seven patients were included, with 26 patients (70%) in the training set and 11 patients (30%) in the validation set. In the latter, all lesions were accurately detected with no false positive resulting in a 100% sensitivity and specificity for tumor detection.

### Segmentation

**Fig 2:**
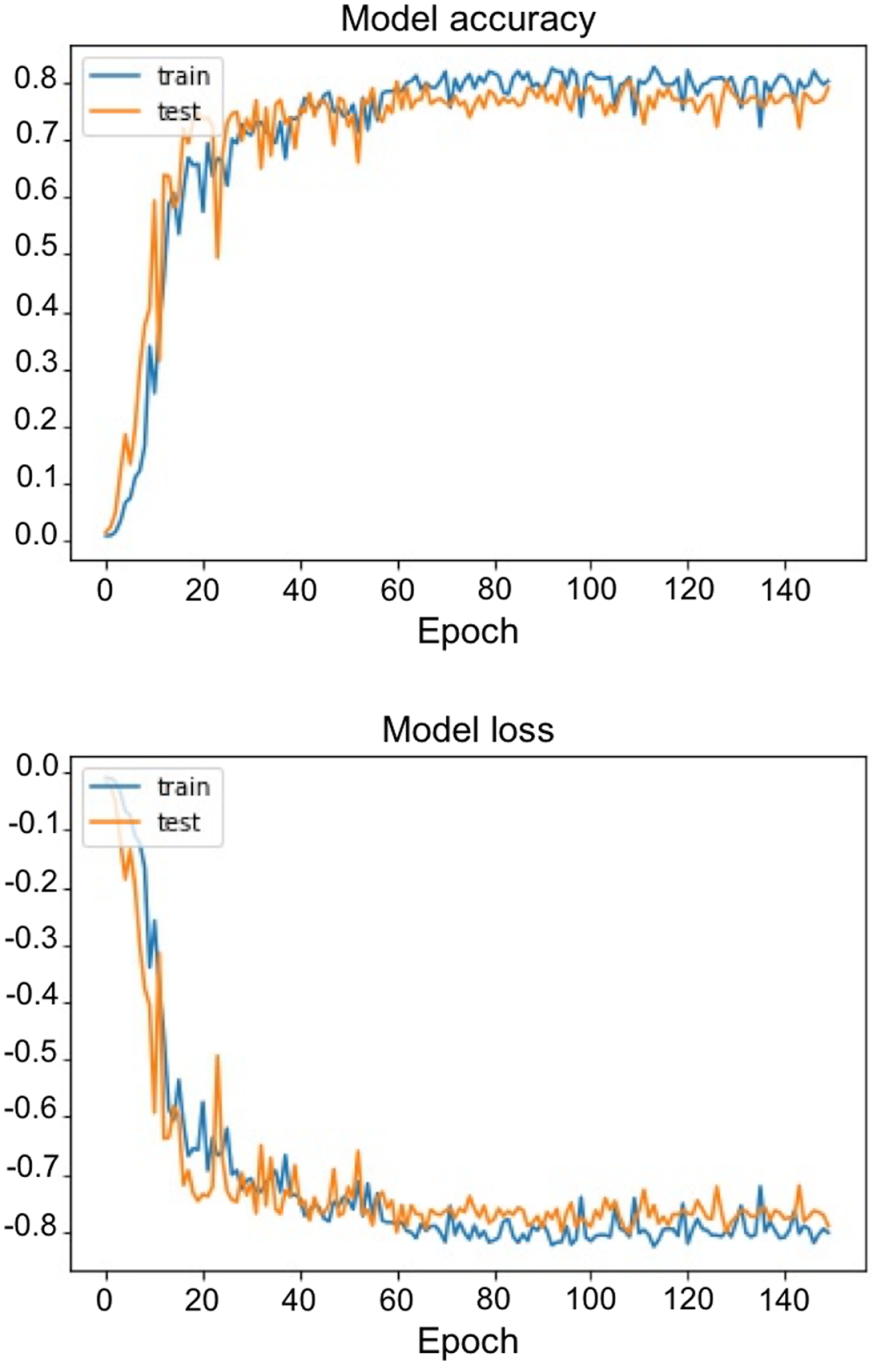
Learning curves with DSC over epochs (upper panel) and loss over epochs (lower panel).

**Fig 3:**
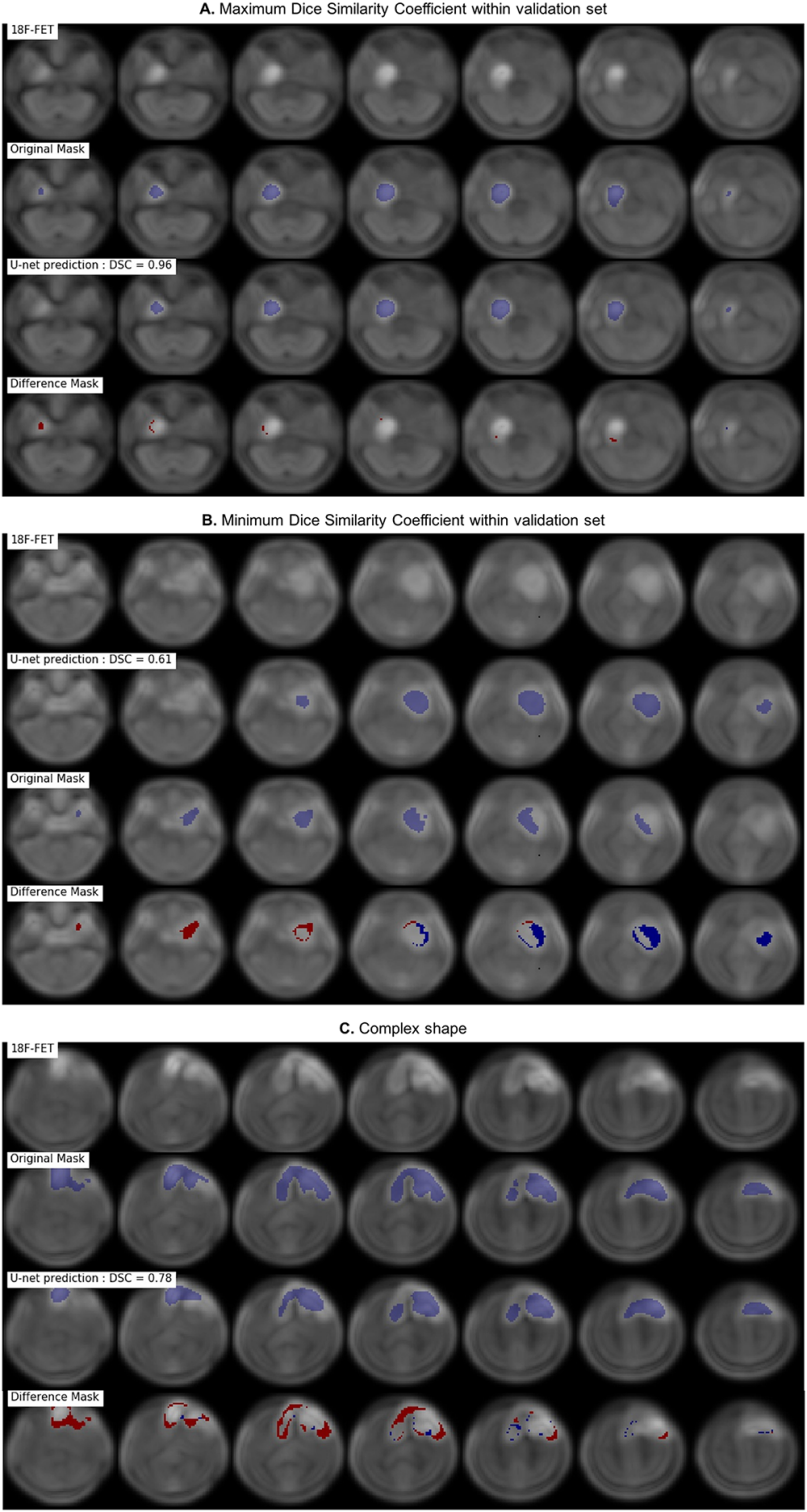
Segmentation examples with for each example: Original resized slices ^18^F-FET (top), original mask (middle up), predicted mask + threshold (middle down) and difference between the predicted and original mask (bottom, with false negative in red and false positive in blue).

After 150 epochs, DSC reached 0.7924 in the training set and 0.7911 in the validation set. Training curves of loss function and DSC curves over epoch can be seen in Fig 2. Morphological dilatation and adaptive thresholding of the predicted U-Net mask leaded to substantial improvement of DSC to 0.8231 (+ 4.1%) in the validation set. At the voxel level, confusion matrix in the validation set is shown in Table 1. It resulted in a 0.88 sensitivity [95% CI, 87.1 to 88.2%], a 0.99 specificity [99.9 to 99.9%], a 0.78 PPV [76.9 to 78.3%] and a 0.99 NPV [99.9 to 99.9%] respectively. These results took into account the large majority of non-tumor voxels. Examples of segmentation are illustrated in Fig 3.

**Table 1:** Confusion matrix at the voxel level in the validation set of 11 patients.

Predicted / truth	True	False
True	10,478	1,426
False	3,025	1,787,261

## Discussion

The aim of the current study was to implement and evaluate a 3D U-Net CNN for brain tumor segmentation of ^18^F-FET PET studies. As shown by the relatively low number of false negative voxels in the validation set resulting in a sensitivity of 88%, it was tried additional resources to catch neighbor voxels that were missed by the U-Net. Therefore, it was tried a region growing approach and a watershed segmentation using random seeds from the predicted U-Net mask. Both did not improve significantly the DSC, and even if it decreased, the number of false negatives impacted negatively the number of false positives (data not shown).

Main limitation of this work includes the relatively small dataset size. Even if some authors argue that small population can be problematic in deep learning (as larger sample size usually leads to improvement of performance [22]), it can be partially corrected by a data-augmentation strategy [23]. Therefore, main data-augmentation strategies using translations or shearing, which may increase the risk of overfitting, should be pondered with either regularization or drop-out. Furthermore, they seem to be the most effective ones for improvement of model performance [24]. Other data-augmentation strategies could also have been employed such as generative adversarial networks where two neural networks compete with each other, the first one (the generator) creates a fictive image volume, and the second one (the discriminator) tries to discriminate whether this volume can “statistically” be drawn from the distribution of the dataset [25]. If the discriminator is fooled and the volume is thought to be a real volume, these images can be used for training. Nevertheless, such approaches would have even more complexes training and methodology that may have lost most of non-deep-learning specialist readers. As part of the preprocessing, a volume resize for computational purposes was performed. It usually leads to a loss of information and may degrade accuracy in performance but this procedure was mandatory due to the limitation of our GPU.

Secondly, it is important to discuss the ground truth generation. For that purpose, it was performed a manual segmentation of ^18^F-FET PET followed by a fixed thresholding as routinely performed for BTV in clinics. There is no consensus on how the adaptive thresholding should be applied. Filss et al. showed that the threshold choice applied had logically a large importance on tumor volume [26]. It is important to note that because of the low resolution and manual segmentation, the absence of thresholding (either fixed or adaptive) would have introduced contour overflow and may also have decreased performance. The choice of using an adaptive thresholding relied both on clinical consideration but also on the fact that two different generations of PET scanners were used in this study, which would have complexified the choice of a fixed threshold. It is probably true that modifications of the ground truth ROI would lead to substantial differences. Indeed, due to the thresholding operations that does not take into account the spatial correlation of voxels, some tumor voxels may have been missed on ground truth as seen in Fig 3B. Some “positive voxels” were clearly within the tumor, but were not considered as such, as the reference mask was thresholded. Regarding the number of false negatives, two main reasons can be hypothesized. They were missed by the U-Net CNN or they were omitted after thresholding. As both original mask and U-Net mask were followed by a fixed thresholding, the different threshold levels between ground truth and predicted mask (one 1.3 times the mean value of 10 cm^3^ in the contralateral hemisphere, the other one 1.3 times the mean value within an hemispheric swap of the predicted mask) may explain this difference. Nevertheless, it was decided to apply this fixed threshold, as it decreased the number of false positive from 3,049 to 1,476 without impacting significantly the number of true positive (from 10,051 to 10,478).

In the future, it is likely that this process of segmentation will be even faster, reproducible and user-friendly with fully automated brain tumor segmentation integrated into clinical routine. These results can be improved at multi-levels. First, some preprocessing operations such as the use of histogram equalization or Contrast Limited Adaptive Histogram Equalization (CLAHE) to improve contrast of these images may better allow the differentiation of background from tumor tissue. Indeed, CLAHE is already used in digital mammograms [27] to enhance contrast for the radiologist eye. Also, the architecture of this CNN can be adapted if more patients are included with deeper layers or cascaded convolutional neural networks [28]. Finally, in the era of hybrid imaging, combining PET and CT/MRI modalities within one examination, it is possible to consider the addition of CT or MRI information using multi-channel deep learning framework. Indeed, even if CT may not have interest (as these tumors are rarely seen on non-enhanced CT), combined PET/MRI systems are now available and may improve multiple tissue characterization and segmentation.

## Conclusion

The current study proposed a full 3D automated approach for brain segmentation from PET images in subjects spanning different glial tumors using a U-Net CNN architecture. To the best of our knowledge, this is the first time that this kind of approach is used with the PET modality. This process was achieved with relatively high performance within the limits of the technique.

## Supporting information

S1 Table Population characteristics. Values are median (25^th^-75^th^ interquartile range) or n (%).
(XLSX)

## References

[1] Dolecek TA, Propp JM, Stroup NE, Kruchko C. CBTRUS Statistical Report: Primary Brain and Central Nervous System Tumors Diagnosed in the United States in 2005–2009. Neuro Oncol. 2012;14 Suppl 5:v1–49. 23095881 10.1093/neuonc/nos218 PMC3480240

[2] Langen K-J, Hamacher K, Weckesser M, Floeth F, Stoffels G, Bauer D, et al O-(2-[18F]fluoroethyl)-L-tyrosine: uptake mechanisms and clinical applications. Nucl Med Biol. 2006;33:287–94. doi: 10.1016/j.nucmedbio.2006.01.002 16631076 10.1016/j.nucmedbio.2006.01.002

[3] Dunet V, Pomoni A, Hottinger A, Nicod-Lalonde M, Prior JO. Performance of 18F-FET versus 18F-FDG-PET for the diagnosis and grading of brain tumors: systematic review and meta-analysis. Neuro Oncol. 2016;18:426–34. doi: 10.1093/neuonc/nov148 26243791 10.1093/neuonc/nov148 PMC4767236

[4] la Fougere C, Suchorska B, Bartenstein P, Kreth F-W, Tonn J-C. Molecular imaging of gliomas with PET: Opportunities and limitations. Neuro Oncol. 2011 1;13:806–19. doi: 10.1093/neuonc/nor054 21757446 10.1093/neuonc/nor054 PMC3145468

[5] Weller M, Pfister SM, Wick W, Hegi ME, Reifenberger G, Stupp R. Molecular neuro-oncology in clinical practice: a new horizon. Lancet Oncol. 2013;14:e370–9. doi: 10.1016/S1470-2045(13)70168-2 23896276 10.1016/S1470-2045(13)70168-2

[6] Sasanelli M, Meignan M, Haioun C, Berriolo-Riedinger A, Casasnovas R-O, Biggi A, et al Pretherapy metabolic tumour volume is an independent predictor of outcome in patients with diffuse large B-cell lymphoma. Eur J Nucl Med Mol Imaging. 2014;41:2017–22. doi: 10.1007/s00259-014-2822-7 24902639 10.1007/s00259-014-2822-7

[7] Jansen NL, Schwartz C, Graute V, Eigenbrod S, Lutz J, Egensperger R, et al Prediction of oligodendroglial histology and LOH 1p/19q using dynamic [18F]FET-PET imaging in intracranial WHO grade II and III gliomas. Neuro Oncol. 2012 1;14:1473–80. doi: 10.1093/neuonc/nos259 23090986 10.1093/neuonc/nos259 PMC3499015

[8] Ceccon G, Lohmann P, Stoffels G, Judov N, Filss CP, Rapp M, et al Dynamic O-(2-18F-fluoroethyl)-L-tyrosine positron emission tomography differentiates brain metastasis recurrence from radiation injury after radiotherapy. Neuro Oncol. 2017;19:281–88. doi: 10.1093/neuonc/now149 27471107 10.1093/neuonc/now149 PMC5463967

[9] Suchorska B, Jansen NL, Kraus T, Giese A, Bartenstein P, Tonn J. Correlation of dynamic 18FET-PET with IDH 1 mutation for prediction of outcome in anaplastic astrocytoma WHO° III independently from tumor vascularisation. J Clin Oncol. 2015;33:15_suppl, 2037–2037.

[10] Pöpperl G, Kreth FW, Mehrkens JH, Herms J, Seelos K, Koch W, et al FET PET for the evaluation of untreated gliomas: correlation of FET uptake and uptake kinetics with tumour grading. Eur J Nucl Med Mol Imaging. 2007;34:1933–42. doi: 10.1007/s00259-007-0534-y 17763848 10.1007/s00259-007-0534-y

[11] Breen SL, Publicover J, De Silva S, Pond G, Brock K, O’Sullivan B, et al Intraobserver and interobserver variability in GTV delineation on FDG-PET-CT images of head and neck cancers. Int J Radiat Oncol Biol Phys. 2007;68:763–70. doi: 10.1016/j.ijrobp.2006.12.039 17379435 10.1016/j.ijrobp.2006.12.039

[12] Hatt M, Lee JA, Schmidtlein CR, Naqa IE, Caldwell C, De Bernardi E, et al Classification and evaluation strategies of auto-segmentation approaches for PET: Report of AAPM task group No. 211. Med Phys. 2017;44:e1–e42. doi: 10.1002/mp.12124 28120467 10.1002/mp.12124 PMC5902038

[13] Shen D, Wu G, Suk H-I. Deep Learning in Medical Image Analysis. Annu Rev Biomed Eng. 2017;19:221–248. doi: 10.1146/annurev-bioeng-071516-044442 28301734 10.1146/annurev-bioeng-071516-044442 PMC5479722

[14] Vander Borght T, Asenbaum S, Bartenstein P, Halldin C, Kapucu O, Van Laere K, et al EANM procedure guidelines for brain tumour imaging using labelled amino acid analogues. Eur J Nucl Med Mol Imaging. 2006;33:1374–80. doi: 10.1007/s00259-006-0206-3 16932934 10.1007/s00259-006-0206-3

[15] Nioche C, Orlhac F, Buvat I. LIFEx: un logiciel gratuit pour caractériser l’hétérogénéité intra-tumorale en imagerie multimodale. Médecine Nucléaire. 2016;40:208.

[16] Nioche C, Orlhac F, Boughdad S, Reuze S, Soussan M, Robert C, et al A freeware for tumor heterogeneity characterization in PET, SPECT, CT, MRI and US to accelerate advances in radiomics. J Nucl Med. 2017;58:1316–1316.

[17] Bette S, Gempt J, Delbridge C, Kirschke JS, Schlegel J, Foerster S, et al Prognostic Value of O-(2-[18F]-Fluoroethyl)-L-Tyrosine-Positron Emission Tomography Imaging for Histopathologic Characteristics and Progression-Free Survival in Patients with Low-Grade Glioma. World Neurosurg. 2016;89:230–9. doi: 10.1016/j.wneu.2016.01.085 26855307 10.1016/j.wneu.2016.01.085

[18] Gempt J, Bette S, Buchmann N, Ryang Y-M, Förschler A, Pyka T, et al Volumetric Analysis of F-18-FET-PET Imaging for Brain Metastases. World Neurosurg. 2015;84:1790–7. doi: 10.1016/j.wneu.2015.07.067 26255241 10.1016/j.wneu.2015.07.067

[19] Popperl G, Gotz C, Rachinger W, Gildehaus F-J, Tonn J-C, Tatsch K. Value of O-(2-[18F]fluoroethyl)-l-tyrosine PET for the diagnosis of recurrent glioma. Eur J Nucl Med Mol Imaging. 2004;31:1464–70. doi: 10.1007/s00259-004-1590-1 15248032 10.1007/s00259-004-1590-1

[20] Ronneberger O, Fischer P, Brox T. U-net: Convolutional networks for biomedical image segmentation. In: International Conference on Medical Image Computing and Computer-Assisted Intervention. Springer; 2015. p. 234–41.

[21] Abraham A, Pedregosa F, Eickenberg M, Gervais P, Mueller A, Kossaifi J, et al Machine learning for neuroimaging with scikit-learn. Front Neuroinform. 2014;8:14 doi: 10.3389/fninf.2014.00014 24600388 10.3389/fninf.2014.00014 PMC3930868

[22] Cho J, Lee K, Shin E, Choy G, Do S. How much data is needed to train a medical image deep learning system to achieve necessary high accuracy? arXiv preprint arXiv:151106348. 2015.

[23] Krizhevsky A, Sutskever I, Hinton GE. Imagenet classification with deep convolutional neural networks. In: Advances in neural information processing systems. 2012 p. 1097–1105.

[24] Wang J, Perez L. The effectiveness of data augmentation in image classification using deep learning. Technical report. 2017.

[25] Goodfellow IJ, Pouget-Abadie J, Mirza M, Xu B, Warde-Farley D, Ozair S, et al. Generative Adversarial Networks. ArXiv e-prints. 2014 Jun 1;1406:arXiv:1406.2661.

[26] Filss CP, Albert NL, Böning G, Kops ER, Suchorska B, Stoffels G, et al O-(2-[18F]fluoroethyl)-l-tyrosine PET in gliomas: influence of data processing in different centres. EJNMMI Res. 2017;7:64 28815478 10.1186/s13550-017-0316-x PMC5559408

[27] Pisano ED, Zong S, Hemminger BM, DeLuca M, Johnston RE, Muller K, et al Contrast Limited Adaptive Histogram Equalization image processing to improve the detection of simulated spiculations in dense mammograms. J Digit Imaging. 1998;11:193–200. doi: 10.1007/BF03178082 9848052 10.1007/BF03178082 PMC3453156

[28] Christ PF, Elshaer MEA, Ettlinger F, Tatavarty S, Bickel M, Bilic P, et al. Automatic liver and lesion segmentation in CT using cascaded fully convolutional neural networks and 3D conditional random fields. In: International Conference on Medical Image Computing and Computer-Assisted Intervention. Springer; 2016. p. 415–423.

